# RETRACTION: miR‐135a Targets SMAD2 to Promote Osteosarcoma Proliferation and Migration

**DOI:** 10.1155/jo/9794048

**Published:** 2026-03-25

**Authors:** Journal of Oncology

RETRACTION: Y. Chen, B. Cai, X. Lian, J. Xu, and T. Zhang, “miR‐135a Targets SMAD2 to Promote Osteosarcoma Proliferation and Migration,” *Journal of Oncology* 2022, no. 1 (2022): 3037348, https://doi.org/10.1155/2022/3037348.

The above article, published online on 13 April 2022 in Wiley Online Library (https://wileyonlinelibrary.com), has been retracted by John Wiley & Sons Ltd. The retraction has been agreed due to multiple figure concerns, as described by *Hoya camphorifolia* and *Actinopolyspora biskrensis* on PubPeer [1].

Specifically, the following concerns were noted:

- Unexpected similarities between Figure 2(d), “HSO–control,” and Figure 2(d), “MG63–inhibitor control”;
- Unexpected similarities between Figure 2(c), “MG63–control,” and Figure 4(c), “MG63–miR‐135a”;
- Unexpected similarities between Figure 4(b), “HSO–miR‐135a,” and Figure 4(b), “HSO–miR‐135a + vector”;
- Unexpected similarities between Figure 2(d), “HSO–inhibitor,” and Figure 4D, “SKOV3–miR‐152+ERBB3“ in [2];
- Unexpected similarities between Figure 2(d), “HSO–control,” and Figure 4D, “HT29–miR‐205‐5p + PTK7” in [3];
- Unexpected similarities between Figure 3(c), “GAPDH–MG63,” and Figure 4C, left “GAPDH” in [4];
- Unexpected similarities between multiple panels in Figure 4(b) and Figure 7(a) in [5];
- Unexpected similarities between multiple panels in Figure 4(d) and Figure 2a in [6].

The publisher has confirmed these concerns independently. The authors contacted the journal to request a correction to Figures 2 and 4 but were later unresponsive to queries regarding the additional concerns. As such, the results and conclusions of this article cannot be considered as reliable and the article is retracted.

## References

[1] Hoya camphorifolia and Actinopolyspora biskrensis, “miR-135a Targets SMAD2 to Promote Osteosarcoma Proliferation and Migration,” *PubPeer* (2023): https://pubpeer.com/publications/9EDE0960A9E9CE08AD98AAF2E73888. 10.1155/2022/3037348 PMC9020941 35466322

[2] Li L.-W. , Xiao H.-Q. , Ma R. , Yang M. , Li W. , and Lou G. , miR-152 Is Involved in the Proliferation and Metastasis of Ovarian Cancer Through Repression of ERBB3, International Journal of Molecular Medicine. (2018) 41, no. 3, 1529–35, 10.3892/ijmm.2017.3324, 2-s2.0-85040984123. 29286064 PMC5819930

[3] Chen S. , Wang Y. , Su Y. et al., miR-205-5p/PTK7 Axis Is Involved in the Proliferation, Migration and Invasion of Colorectal Cancer Cells, Molecular Medicine Reports. (2018) 17, no. 5, 6253–60, 10.3892/mmr.2018.8650, 2-s2.0-85044748812. 29488611 PMC5928600

[4] Zhou J. , Dai W. , and Song J. , miR-1182 Inhibits Growth and Mediates the Chemosensitivity of Bladder Cancer by Targeting hTERT, Biochemical and Biophysical Research Communications. (2016) 470, no. 2, 445–52, 10.1016/j.bbrc.2016.01.014, 2-s2.0-84955445000. 26772886

[5] Xu X. , Zong Y. , Gao Y. et al., RETRACTED: VEGF Induce Vasculogenic Mimicry of Choroidal Melanoma Through the PI3k Signal Pathway, BioMed Research International. (2019) 2019, no. 1, 10.1155/2019/3909102, 2-s2.0-85069797690. PMC6657640 31380420

[6] Hong Q. , Li X.-D. , Xie P. , and Du S.-X. , All-Trans-Retinoic Acid Suppresses Rat Embryo Hindlimb Bud Mesenchymal Chondrogenesis by Modulating HoxD9 Expression, Bioengineered. (2021) 12, no. 1, 3900–3911, 10.1080/21655979.2021.1940613. 34288810 PMC8806522

